# The impact of the COVID-19 pandemic on refugees and asylum seekers in Greece: A retrospective analysis of national surveillance data from 2020

**DOI:** 10.1016/j.eclinm.2021.100958

**Published:** 2021-07-01

**Authors:** Elias Kondilis, Dimitris Papamichail, Sophie McCann, Elspeth Carruthers, Apostolos Veizis, Miriam Orcutt, Sally Hargreaves

**Affiliations:** a Laboratory of Primary Health Care, General Medicine and Health Services Research, School of Medicine, Aristotle University of Thessaloniki, Thessaloniki, Greece; b Department of Public Health Policy, University of West Attica, Athens, Greece; c Lancet Migration global collaboration to advance migration health, Institute for Global Health, University College London, 30 Guilford Street, London WC1N1EH, UK; d Institute for Global Health, University College London, 30 Guilford Street, London WC1N1EH, UK; e Lancet Migration global collaboration to advance migration health, Institute for Global Health, University College London, 30 Guilford Street, London WC1N1EH, UK; f INTERSOS Hellas, 31 Ermou Street, Thessaloniki 546 24, Greece; g The Migrant Health Research Group, Institute for Infection and Immunity, St George's, University of London, London, UK

**Keywords:** COVID-19, Migrant, Health inequalities, Vaccination, Refugee, Greece

## Abstract

**Background:**

Migrants globally, including refugees and asylum seekers, have experienced adverse clinical and socioeconomic impacts of the COVID-19 pandemic. For approximately 56,000 refugees and asylum seekers in Reception and Identification Centers (RICs) and Reception Sites (RS) in Greece, living in severely substandard living conditions, prevention measures have been impossible with limited provision in terms of routine testing, surveillance, and access to healthcare. These migrant populations have experienced prolonged lockdowns and restricted movement since the pandemic began. We aimed to assess the impact of COVID-19 on refugees and asylum seekers in reception facilities in Greece and explore implications for policy and practice.

**Methods:**

A retrospective analysis of policy documents and national surveillance data was conducted to identify COVID-19 outbreaks and estimate incidence among asylum seekers and refugees residing in these camps during the first 9 months of the epidemic in Greece (26th February – 15th November 2020). Incidence proportion (IP) of COVID-19 confirmed cases was calculated for three population groups (refugees and asylum seekers in RICs, refugees and asylum seekers in RSs, and the general population in Greece) during three time periods (first wave, second wave, and overall across the 9-month period).

**Findings:**

Twenty-five COVID-19 outbreaks were identified in refugee and asylum seeker reception facilities, with 6 (85.7%) of 7 RICs and 18 (56.3%) of 32 RSs reporting at least one outbreak during the study period. The overall 9-month COVID-19 IP among refugee and asylum seeker populations residing in RSs on the Greek mainland was 1758 cases per 100,000 population; in RICs the incidence was 2052 cases per 100,000 population. Compared to the general population the risk of COVID-19 infection among refugees and asylum seekers in reception facilities was 2.5 to 3 times higher (*p*-value<0.001). The risk of acquiring COVID-19 infection was higher among refugee and asylum seeker populations in RSs on the Greek mainland (IP ratio: 2.45; 95% CI: 2.25–2.68) but higher still among refugee and asylum seeker populations in RICs in the Greek islands and the land border with Turkey (IP ratio: 2.86; 95% CI: 2.64–3.10), where living conditions are particularly poor.

**Interpretation:**

We identified high levels of COVID-19 transmission among refugees and asylum seekers in reception facilities in Greece. The risk of COVID-19 infection among these enclosed population groups has been significantly higher than the general population of Greece, and risk increases as living conditions deteriorate. These data have immediate implications for policy and practice. Strategies are now needed to ensure refugee and asylum seeker populations are included in national response plans to reduce transmission in at-risk groups for COVID-19, alongside inclusion in plans for COVID-19 vaccine roll out.

**Funding:**

None.

**Research in context**

**Evidence before this study**

There is very limited peer-reviewed and grey literature available on the impact of the COVID-19 pandemic refugee and asylum seekers in Greece, particularly those living in closed settings such as reception centres. Peer reviewed literature available at global level on the impact of the COVID-19 on migrants is also limited, with only one systematic review by Hayward et al. reporting on clinical outcomes and risk factors for COVID-19 among migrant populations in high-income countries. This systematic review documents potentially higher levels of transmission in closed settings, including migrant reception and detention centres, and suggests migrants in general may be over-represented in cases and deaths.

**Added value of this study**

Our study explores the impact of COVID-19 on refugees and asylum seekers residing in reception facilities in Greece, and its implications for policy and practice. Our data suggest that these overcrowded camps may have resulted in increased infection risk for COVID-19. For the first 9-month epidemic period in Greece (first and second wave) our findings show that refugees and asylum seekers in these reception facilities have almost a 2.5 to 3 times higher COVID-19 incidence compared to the general population (p-value <0.001), with highest risk in migrant populations in reception facilities on the Greek islands and at the Greek-Turkish border, where living conditions are considerably worse.

**Implications of all the available evidence**

Our data highlights that refugee and asylum seeker populations residing in reception and identification centres and reception sites in Greece are at higher risk of acquiring SARS-CoV-2 infection. Going forward, an urgent focus must be placed on improving living conditions in these facilities, decongesting those that operate beyond their capacity, reinforcing epidemiological surveillance, testing strategies and data transparency, and ensuring free, comprehensive and universal access to care. Refugees and asylum seekers in closed settings need to be urgently prioritised in the COVID-19 vaccine roll-out.

Alt-text: Unlabelled box

## 1 Introduction

Migrants – including marginalized groups such as asylum seekers, refugees, low-wage labor migrants, and undocumented migrants – globally have experienced adverse clinical and socioeconomic impacts of the COVID-19 pandemic [1]. Pre-existing structural inequalities have been exacerbated by the COVID-19 pandemic, with migrants already experiencing barriers to accessing healthcare even before the outbreak of COVID-19 and often legal exclusion from European health systems [2], alongside high levels of poverty and deprivation. The discrimination they may face, and their fear and lack of trust in authorities means several thousands of migrants in Europe do not engage in health systems [3], an important concern during a global pandemic. Where migrants do feature in public health policy, it is often in the context of health security and infection control [4]. They may face barriers, therefore, to accessing COVID-19 testing, treatment and vaccination, even when legally entitled, and xenophobic discourse around migrants and infectious disease may further alienate them from health systems [5,6].

Greece is a major entry point for people seeking safety in Europe, and since the early 2000s hundreds of thousands of asylum seekers have crossed into Greece by land and sea. In 2015, over 800,000 people arrived in Greece, the majority fleeing the war in Syria, as well as conflicts and violence in Afghanistan, Iraq and some sub-Saharan African countries [7]. As a result of the EU-Turkey agreement, since March 2016 [8], thousands of men, women and children have been trapped on five Aegean Island Reception and Identification centres (RICs) pending their asylum application process, and in one RIC on the Greek-Turkish land border. In addition, several thousand asylum seekers and refugees are also now accommodated in Reception Sites (RSs) on the Greek mainland, comprising 32 International organization for Migration (IOM)-run ‘open camps’ and in hotels and apartments (although data from these settings is unclear) throughout the Greek mainland [9], as well as almost 26,000 accommodation spaces on the Greek islands and mainland for vulnerable asylum seekers and refugees provided by the United Nations Refugee Agency (UNHCR) [10]. As of December 2020, around 120,000 refugees, asylum seekers and migrants were estimated to be residing on the Greek islands and the mainland [11], and of those, 56,000 asylum seekers and refugees are in the RICs and IOM-run RSs. Women and children comprise around 67% of the total population living in IOM-run RS with the majority of residents coming from Afghanistan, Syria and Iraq [12]. Women and children comprise approximately 49% of the total population living in the island RICs with the majority of residents coming from Afghanistan, Syria, the Democratic Republic of Congo and Somalia [13].

Both the RICs and mainland RSs are characterized by severely substandard living conditions, poor sanitation and a lack of access to adequate – or sometimes any – healthcare, creating significant risk for their inhabitants in the event of an outbreak of infectious disease [14], with conditions on the islands and Greek-Turkish land border considerably worse than the mainland with higher levels of overcrowding. Social distancing and frequent handwashing have been virtually impossible during the COVID-19 pandemic: residents must often queue in close proximity in order to receive food and other essential items and self-isolation away from other individuals is not feasible due to the cramped conditions [15,16]. Asylum seekers and refugee populations have also experienced prolonged lockdowns and restricted movement since the pandemic began. These populations in Greece continue to face multiple administrative, geographical, societal and legal barriers to accessing mainstream healthcare [16]. The European center for Disease Prevention and Control (ECDC) has suggested that such settings pose a high-risk for COVID-19 transmission and these populations may need to be prioritized for COVID-19 vaccine roll out [[17], [18], [19]].

The COVID-19 epidemic in Greece started on 26th February 2020, and during the first epidemic wave Greece managed to keep COVID-19 infections, Intensive Care Units hospitalized cases and deaths at relatively low levels compared to other European countries [20]. However, an exponential increase in COVID-19 cases and deaths was subsequently recorded between November and early December 2020 and a second national lockdown was then imposed [21]. The extent to which the COVID-19 pandemic has impacted on refugees and asylum seekers housed in RICs and RS, however, has not to date been fully investigated. We therefore aimed to explore and assess the impact of COVID-19 on refugees and asylum seekers residing in reception facilities in Greece, and its implications for policy and practice.

## 2 Methods

We conducted a retrospective observational study analyzing secondary surveillance data and policy documents from multiple sources in order to: (a) identify the number of COVID-19 outbreaks and cases in refugee and asylum seeker reception facilities in Greece and (b) compare the incidence of COVID-19 among refugee and asylum seeker populations residing in Greek RICs and RSs to the incidence among the Greek host population. We analyzed data from the first nine months of the epidemic in Greece (26th February until 15th November 2020), which was divided into two distinct time periods: the first epidemic wave of COVID-19 in the country (26th February until 30th June 2020) and part of the second wave period (1st July to 15th November 2020). Our study period starts from 26th February 2020 when the first COVID-19 case was confirmed in Greece and ends on 15th November 2020 which, at the time of embarking on this study, was the most up to date data available regarding COVID-19 cases among refugee and asylum seekers in Greece.

We focused on two particular migrant groups: ‘asylum seekers’ who are individuals with an active asylum claim pending, and ‘refugees’ who have been granted international protection and are recognized refugees in Greece with their claims processed, residing in two types of accommodation in Greece: (i) seven RICs – 6 RICs on 5 specific Greek islands (including Moria and Kara Tepe facilities on Lesvos) and one RIC on the Evros border; and (ii) 32 IOM-run RSs on the Greek mainland.

### 2.1 Data sources

We identified a range of data sources for data pertaining to cases in both the general population, RICs, and RSs. The total number of COVID-19 laboratory confirmed cases in the overall population in Greece were derived from the COVID-19 epidemiological surveillance report published daily by the Hellenic Public Health organization (NPHO), which is the national center for disease control and prevention [22]. Numbers of COVID-19 laboratory confirmed cases among refugees and asylum seekers residing in RICs and RSs were derived from the COVID-19 epidemiological surveillance reports in points of care for refugees/migrants/asylum seekers published on a weekly basis by the same organization [23]. The reporting of cases in these datasets for the refugees and asylum seekers residing in RICs and RSs is cumulative on a weekly basis and COVID-19 infections are disaggregated only by type of facility (RICs versus other RSs).

Official information about the number and specific location of COVID-19 outbreaks in RICs and RSs in Greece is not publicized by Greek authorities [24]. Media and non-governmental organization (NGO) reports have occasionally revealed cases of large outbreaks in refugee and asylum seeker camps in Greece [25], but these reports present data and information on an ad-hoc basis. In April 2020 the Greek Ministry of Migration and Asylum introduced the “Agnodiki plan”, which is an epidemic management plan activated in cases of outbreaks in refugee and asylum seeker reception centres and facilities [26]. The “Agnodiki plan” was activated by a Ministerial Decree published on the Government Gadget and publicized on the Greek COVID-19 legislation database. We reviewed a total of 891 pieces of legislation included in this database [27], we identified all “Agnodiki plan” activation Ministerial Decrees issued by the Greek Ministry of Migration and Asylum from 27th February to 25th October 2020 with the aim of identifying outbreaks and cases in RICs and RSs involving refugees and asylum seekers. These Ministerial Decrees provide information about the exact location of an outbreak in a reception center or facility, outbreaks that triggered subsequently the activation of the Ministerial plan.

### 2.2 Ethics approval

No ethics approval was required; all the data sources are anonymous and freely available online.

### 2.3 Statistical analysis

Incidence proportion (IP) of COVID-19 confirmed cases was calculated for our three study population groups (refugees and asylum seekers populations in RICs, refugees and asylum seekers in RSs and general population in Greece) during three time periods (first wave, second wave, and overall across the 9-month period). IP reflects the new COVID-19 cases during a specific time period divided by the population at risk at the beginning of the relevant period. Because we anticipated that the size of refugee and asylum seeker populations residing in RICs and other RSs would change over time, we calculated the mean population at risk based on reports on the number of refugees and asylum seekers in RICs provided on a weekly basis by the Hellenic Ministry of National Defense [28], and reports on the number of refugees and asylum seekers in other RSs provided on a monthly basis by the IOM [29]. Information regarding the Greek population was based on the latest census data [30].

Total number of COVID-19 cases in the general population was calculated by combining the two publicly available data sets on COVID-19 confirmed cases [31,32], and after subtracting the total number of cases among refugees and asylum seekers from the total number of cases in Greece. In order to compare IP in each of the two refugee and asylum seeker population groups to the IP of the general population in Greece (control group), we calculated the incidence proportion ratio (IPR) and the relevant 95% Confidence Intervals (CI). *P-value* less than 0.05 was considered statistically significant. Data were extracted by EK and duplicate data extraction was done by DP. The analysis was carried out by DP and EK using STATA (version 11) and OpenEpi [33], in order to calculate IP and IPR and 95% CI's, respectively.

### 2.4 Role of the funding source

None. EK accessed the database and EK, MO, SH, and AV took the decision to submit the paper for publication.

## 3 Results

During our study period (26th February – 15th November 2020) 77,527 COVID-19 confirmed cases were reported among the general population in Greece (approximately 10.8 million people), most of which (74,315 cases; 95.9%) were observed during the second epidemic wave, and 3212 (4.1%) during the first.

### 3.1 COVID-19 outbreaks in refugee and asylum seeker RICs and RSs: first and second wave

**Fig. 1:**
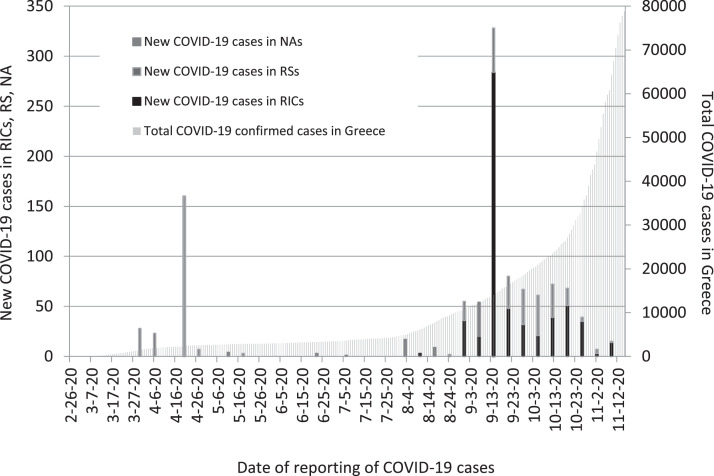
COVID-19 confirmed cases among refugee and asylum seeker populations residing in Reception Identification Centers and other Reception Sites in Greece (26 Feb 2020 – 15 Nov 2020). RICs=Reception and Identification Centers; RSs=Reception Sites; NAs=New Arrivals.

During the first epidemic wave (February to June 2020) three outbreaks were reported in refugee and asylum seeker RSs on the Greek mainland (in Ritsona, Malakasa and Kranidi reception sites). All these outbreaks took place between late March and late April 2020, when the first epidemic wave was partly under control and overall cases in the country were rapidly declining (Fig. 1). Importantly, we found that the identification and reporting of all three initial outbreaks was incidental and related to individual residents testing positive for COVID-19 in external sites such as hospitals, and not as part of government routine testing within the RSs. Among the three observed outbreaks during the first epidemic wave the largest outbreak took place in the Kranidi reception site, with a total of 157 COVID-19 confirmed cases. In Ritsona RS a total of 41 positive cases were detected, and in Malakasa RS 23 positive cases were detected.

Throughout the second epidemic wave, 22 COVID-19 outbreaks were detected in both RICs and RSs. Among these 22 outbreaks, five (22.7%) took place in the RICs on the Greek islands, one (4.5%) in the RIC on the Greek-Turkish border, and 16 (72.7%) outbreaks occurred in other RSs on the Greek mainland (Table 1). All these outbreaks took place between early August and early October, at the start of the second epidemic wave and before the exponential increase in COVID-19 cases and the ensuing introduction of the second national lockdown in early November (Fig. 1, Table 1). During the second epidemic wave COVID-19 outbreaks were detected in six (85.7%) of 7 of the operating RICs, and in 16 (50%) of 32 of the other operating RCs in the country. Among the 22 observed outbreaks during the second wave, the largest one took place in the Kara Tepe tent camp on Lesvos Island in mid-September 2020; however, the final number of detected COVID-19 cases are not reported, with no official data having yet been made available.

**Table 1:** COVID-19 outbreaks in reception identification centers and other reception sites during the second wave in Greece (July – Nov 2020).

Date of outbreak	COVID-19 outbreaks by type of refugee facility and location
02.08.20	RIC Evros *(Greek NorthEast border)*
13.08.20	RIC Chios *(Greek island)*
14.08.20	RS Drama *(Greek mainland)*
02.09.20	RIC Lesvos *(Greek island)*, RS Ritsona *(Greek mainland)*
07.09.20	RS Schisto, RS Elaionas, RS Malakasa *(Greek mainland)*
11.09.20	RS Alexandreia, RS Veroia, RS Katerini, RS Polykastro *(Greek mainland)*
15.09.20	RIC Samos, RIC Leros *(Greek islands)*
18.09.20	RIC Kara Tepe - Lesvos *(Greek island)*, RS Koutsochero *(Greek mainland)*
25.09.20	RS Serres, RS Thiva *(Greek mainland)*
02.10.20	RS Skaramagas *(Greek mainland)*
05.10.20	RS Ioannina *(Greek mainland)*
06.10.20	RS Kilkis *(Greek mainland)*
07.10.20	RS Doliana *(Greek mainland)*

Notes: RICs (Reception and Identification Centers), RSs (Reception Sites).

### 3.2 COVID-19 incidence proportion in refugee and asylum seeker populations in RICs and RSs in Greece

Throughout both waves of the epidemic in Greece, 1106 COVID-19 confirmed cases have been reported amongst the refugee and asylum seeker population residing in reception facilities; 228 cases (20.6%) were reported during the first epidemic wave and 878 (79.4%) during the second (Table 2). This pattern follows the overall development of the epidemic in Greece and reflects the severity of the second epidemic wave in the country. Among the total 1106 COVID-19 confirmed cases, 587 (53.1%) were detected among refugee and asylum seekers residing in RICs, 485 (43.9%) among refugees and asylum seekers residing in other RSs on the Greek mainland and 34 (3.1%) among newly arrived asylum seekers in the Greek Islands during the epidemic (Table 2).

**Table 2:** Confirmed COVID-19 cases among refugees and asylum seekers residing in reception facilities, by epidemic period and by type of facility, Greece.

	1st Epidemic Wave (26 February – 30 June 2020)		2nd Epidemic Wave (1 July – 15 November 2020)		Overall epidemic period (26 February – 15 November 2020)	
	N cases	%	N cases	%	N cases	%

Migrants in RICs	0	0.0	587	66.9	587	53.1
Migrants in RSs	221	96.9	264	30.1	485	43.9
Migrants, new arrivals	7	3.1	27	3.1	34	3.1
Total	228	100.0	878	100.0	1106	100.0

Note: RICs (Reception and Identification Centers), RSs (Reception Sites) (B) COVID-19 cases among refugees and asylum seekers refer only to cases reported in the 7 RICs and the 32 IOM-run RSs operating in the country.

The overall 9-month COVID-19 incidence proportion among refugee and asylum seeker populations residing in RSs on the Greek mainland was 1758 cases per 100,000 population, while for people residing in RICs the incidence increases to 2052 cases per 100,000 population (Table 3).

**Table 3:** COVID-19 Incidence Proportion in Reception Identification Centers and other Reception Sites, by epidemic period in Greece.

	1st Epidemic Wave (26 February – 30 June 2020)			2nd Epidemic Wave (1 July – 15 November 2020)			Overall epidemic period (26 February – 15 November 2020)		
	Population at risk	COVID-19 Incidence Proportion (IP) per 100,000 population	IP Ratio (95% CI)	Population at risk	COVID-19 Incidence Proportion (IP) per 100,000 population	IP Ratio (95% CI)	Population at risk	COVID-19 Incidence Proportion (IP) per 100,000 population	IP Ratio (95% CI)
Greek population	10,816,286	30	Ref.	10,813,714	687	Ref.	10,816,286	717	Ref.
Migrants in RICs (Greek islands and border)	35,435	0	Not applicable	21,772	2696	3.92 (3.62–4.25)	28,603	2052	2.86 (2.64–3.10)
Migrants in RSs (Greek mainland)	26,910	821	27.66 (24.14–31.68)	28,428	936	1.36 (1.21–1.54)	27,584	1758	2.45 (2.25–2.68)

Notes: (A) RICs (Reception and Identification Centers), RSs (Reception Sites), Ref. (Reference Group), (B) the Incidence Proportion Ratio refers to the Incidence Proportion of COVID-19 infections among migrants residing in RICs or RS compared to the control group (which is the general Greek population) (C) COVID-19 cases among refugees and asylum seekers refer only to cases reported in the 7 RICs and the 32 IOM-run RSs operating in the country.

During the first epidemic wave the risk of infection was almost 28 times higher in refugees and asylum seekers residing in the 32 IOM-run RSs on the Greek mainland compared to the general population (IPR: 27.66; 95% CI: 24.14–31.6), a finding likely related to the three outbreaks that were recorded in mainland RSs during the first wave. Throughout the second epidemic wave the risk of infection was almost 4 times higher in refugees and asylum seekers residing in RICs on Greek islands and the Evros border compared to the general population (IPR: 3.92; 95% CI: 3.62–4.25). This is linked to the outbreaks that were recorded in almost all RICs (with the only exception being the RIC in Kos Island) during the second wave.

For the overall 9-month epidemic period in Greece our findings show that refugee and asylum seeker population groups in RICs and RSs have almost a 2.5 to 3 times higher COVID-19 incidence compared to the general population (*p*-value<0.001). The risk of infection (that is the incidence of COVID-19 symptomatic and asymptomatic infections regardless their severity and clinical outcomes) increases between the general population and refugee and asylum seeker populations in RSs on the Greek mainland (IPR: 2.45; 95% CI: 2.25–2.68), and is highest in migrant populations in RICs on the islands and Greek-Turkish border, where living conditions are considerably worse (IPR: 2.86; 95% CI: 2.64–3.10) (Table 3).

## 4 Discussion

We identified high levels of COVID-19 transmission among asylums seekers and refugees in RICs and RSs on the Greek mainland and surrounding islands. The risk of infection among these enclosed population groups has been significantly higher than the general population of Greece, and risk increases as living conditions deteriorate and overcrowding increases. These data have immediate implications for policy and practice.

There were several identified study limitations. First, there is poor epidemiological surveillance and data collection in Greece and we were reliant on national statistics which were limited in scope and access. The likely under-reporting among refugees and asylum seekers not presenting to healthcare providers means we do not have a clear picture of the rates of COVID-19 amongst this population. In addition, the datasets we had access to provided no information on health outcomes in COVID-19 cases involving refugees and asylum seekers, including hospitalisations in COVID-19 clinics, hospitalisations in Intensive Care Units (ICUs), or deaths and additionally no information regarding age and sex distribution of COVID-19 cases among refugees and asylum seekers was publicly available.

The refugee and asylum seeker population tends to be younger, with one NGO report stating that over 60% of refugees are under 30 years old [34]. Children comprise approximately 28% of the populations in the RICs and approximately 44% of the population in the IOM-run RSs [12,13]. At the start of the pandemic 2400 elderly people and those at-risk living in the island RICs were identified to be moved to safer accommodation [35]. However, given the lack of information on the exact age structure of the refugee and asylum seeker population residing in RICs and RSs in Greece and the lack of information on the age and sex distribution of COVID-19 confirmed cases among these population groups, it is unclear whether age did play a significant role in the increased infection rate amongst asylum seekers and refugees compared with the national population.

Living conditions in the RICs and RSs make adherence to COVID-19 public health guidance and prevention very challenging, and most likely explain the higher transmission our data show in these settings. Physical distancing and hygiene measures (e.g. handwashing) were encouraged by authorities at the start of the pandemic, but due to the severe overcrowding of the RICs [36] and the substandard living conditions and inadequate sanitation services, these were impossible to follow, increasing their vulnerability to COVID-19. For example, following a fire that destroyed Lesvos Island's Moria RIC in September 2020, the refugees and asylum seekers were forced to move into the Kara Tepe tent-camp with no access to food, medical or psychological care, water points, showers or toilets thus lacking basic preventive measures for COVID-19 [37,38]. Even by the end of the second wave in the new Kara Tepe camp there were limited pandemic protections in place and most residents still had little access to toilets and washing facilities, with no hot water despite the winter weather [39]. Those in quarantine on Samos were reported by Médecins Sans Frontières to be living in ‘unacceptable and dangerous’ conditions, with some in filthy containers with no access to running water or toilets and forced to sleep on the floor [40].

Asylum seekers and refugees in Greece face multiple administrative and legal barriers to accessing healthcare, further disadvantaging them in the context of COVID-19. Healthcare provision for those living in RICs and RSs is inadequate, with long wait time to see a doctor if there is one present at all, and often with limited options for swiftly accessing secondary care [16,41]. Furthermore, there is also a degree of xenophobia towards the refugee and asylum seeking population [42], which impacts the provision of healthcare and likely affects health seeking behavior, which may have increased the infection risk as well as leading to under-reporting of suspected COVID-19 cases.

Despite the ECDC's recommendation to consider ‘measures to decongest and evacuate residents’ in these enclosed settings if distancing and other risk mitigation measures cannot be implemented [19], and the Greek government's pledges to decongest and improve conditions, authorities are yet to fully addressed these issues, which is an urgent next step in limiting outbreaks. The government had pledged to move 2400 high-risk individuals (elderly and those with underlying conditions) living in RICs to safe accommodation, but as of 14th December 2020 there were still 207 ‘high risk’ individuals remaining in the five island facilities [43]. Although data are lacking on clinical outcomes in refugees and asylum seekers residing in RICs and RSs, some early data suggest that this group may be disproportionately represented in hospitalized cases in some areas near migrant RICs and RSs. The President of the Panhellenic Association of Public Hospital Employees confirmed on 17th September 2020 that almost half the COVID-19 patients hospitalized in the mainland Attica region were refugees and asylum seekers from RSs and homeless migrants from Athens [44]. The Minister of Health noted they could not be discharged and returned to the RSs or to the streets due to the poor living conditions and overcrowding [45].

Although Greece was swift to impose early nationwide public health restrictions, the lockdown measures and mass quarantining were applied more stringently and for longer periods of time to refugees and asylum seekers in RICs and RSs. The government declared these targeted measures to be in the public interest to ‘“limit the spread of COVID-19 in areas of overcrowding…” [46], despite no positive COVID-19 cases in the RICs until mid-August. Similarly, one mainland RS was put into preventative quarantine despite no detected positive cases of COVID-19 [16]. These extreme lockdown extensions contravened WHO [47] and ECDC guidelines [19] and restricted people's movement in such conditions with no likelihood of being able to respect basic COVID-19 preventative measures. Our data suggest that these restrictive policies may have contributed to their increased infection risk for COVID-19. Data from other countries housing refugees and asylum seekers in closed settings, such as reception and detention centres, aligns with our findings. For example, COVID-19 outbreaks have been reported in: an open center in Malta [48], and reception centres in Germany [49], the Netherlands [50], and Italy [51]. In Germany, in temporary housing for asylum seekers and refugees, 42 centres were affected and 30 of 42 centres were put under mass quarantine [49].

Despite calls for inclusion of refugees and asylum seekers in the COVID-19 response from multi-laterals such as WHO, UNHCR, ECDC and IOM [19,52], and academic organizations such as Lancet Migration [53], Greek authorities have consistently failed to integrate refugees and asylum seekers into national prevention and response plans and disease surveillance systems, and no coherent medical response plans have been put in place in any of the island RICs. Even before the COVID-19 pandemic the impact of poor living conditions was already driving a health crisis on the Greek islands [54] and healthcare access for refugees and asylum seekers in Greece had been a continual challenge since 2015 [55]. After nine months of the pandemic, the Greek authorities have not established an effective and comprehensive testing and contact tracing system for refugees and asylum seekers in Greece, despite having a functioning system for the general population. It is our view that the inadequate testing and the absence of contact tracing in refugee and asylum seeker reception facilities has led to an underestimation of the true incidence rate amongst the refugee and asylum seeker populations. Greek authorities face serious challenges in collecting and presenting timely and comprehensive data on the development of the epidemic in the country [24], and the lack of data on clinical outcomes in refugees and asylum seekers specifically in Greece (specifically hospitalizations and deaths) needs urgently rectifying, though this is a reported issue in several European countries at the current time [1].

In order to ensure inclusion of refugees and asylum seekers in the COVID-19 response, a greater emphasis must also be placed on developing culturally and linguistically adapted information about COVID-19 prevention measures and vaccination. In a rapid review public health communication targeting migrant populations during the COVID-19 pandemic conducted in June 2020, for example, only half of the European member states had translated their COVID-19 risk communications into one migrant language; and no government produced risk communications on COVID-19 prevention targeting people in refugee camps or informal settlements, leaving migrants excluded from national health system responses [56].

Our data quantitatively shows the greater risk of COVID-19 infection among refugee and asylum seeker populations residing in substandard living conditions. Acknowledging this higher risk is of crucial policy importance. Going forward, all enclosed population groups need to be urgently included and prioritized in the national vaccination program, without any exception. Greece should follow the example of the Republic of Serbia which, in late March became one of the first European countries to begin vaccinating its refugee, asylum seeker and other migrant populations living in reception centres and has included these populations in their national vaccination plan [57]. However, given the time needed in order to achieve herd immunity through vaccination, Greek authorities should concurrently urgently focus on improving living conditions in these facilities, decongesting those that operate beyond their capacity, reinforcing epidemiological surveillance, testing strategies and data transparency, and ensuring free, comprehensive and universal access to care without any discrimination. In this direction, we propose the following key recommendations and next steps:

(1) In order to reduce the risk of transmission of COVID-19 and to protect the right to health for all, the Greek government should urgently improve living conditions in all reception facilities including the provision of adequate sanitation and hygiene services, dignified and safe accommodation where COVID-19 prevention measures can be followed.
(2) Interagency guidance on protection and response to COVID-19 issued by multiple agencies in the first half of 2020 should be adhered to a by all governments [58], [59], [60]. In addition, access to routine testing for COVID-19, treatment, and COVID-19 vaccinations as well as other vaccines should be ensured for refugees, asylum seekers (and other migrants) outside of mainstream health systems in Greece and across Europe, especially in enclosed settings such as reception and detention centres. Comprehensive testing and tracing amongst refugee and asylum seeker populations in Greek RICs and RSs needs to be scaled up, and refugees and asylum seekers meaningfully included in national prevention and response plans to COVID-19. This will involve developing tailored and targeted public health messaging being mindful of the specific needs of these populations.
(3) Restrictive public health measures, such as lockdowns and mass quarantining, should only be applied where proportionate to public health need, not as a blanket measure for certain population groups such as migrants. Quarantine and lockdown measures should be applied equally to all without discrimination; healthcare, social and psychosocial support, and basic needs as food, water and other essentials should be provided to those in quarantine; and mass quarantine avoided where possible, in line with ECDC and WHO guidelines.
(4) Epidemiological surveillance systems must be strengthened to better record COVID-19 cases, hospitaliations, and deaths involving refugees, asylum seekers, and other migrants, in Greece and Europe-wide to inform the public health response going forward and implications for COVID-19 vaccine roll out. A rigorous system for public data sharing should be established, with an emphasis on transparency and timeliness.

## Funding

None

## Data sharing statement

No individual participant data was used. All data sources for data pertaining to cases in both the general population, RICs, and RSs, are freely available online. Any query on the manuscript can be submitted to the corresponding authors.

## Declaration of Competing Interest

MO is Executive Director of Lancet Migration: global collaboration to advance migration health. All other authors declare no conflicts of interest.

## References

[1] Hayward S., Deal A., Cheng C., Crawshaw A., Orcutt M. (2021). Clinical outcomes and risk factors for COVID-19 among migrant populations in high-income countries: a systematic review. J Migr Health.

[2] I. Abubaker, R. Aldridge, D. Devakumar, M. Orcutt, R. Burns, M. Barreto, et al. The UCL-lancet commission on migration and health: the health of a world on the move. 2018. doi: 10.1016/S0140-6736(18)32114-7. [Accessed 9th February 2021]

[3] Lebano A., Hamed S., Bradby H., Gil-Salmerón D.-.F.E. (2020). A. Migrants’ and refugees’ health status and healthcare in Europe: a scoping literature review. BMC Public Health.

[4] Zimmerman C., Kiss L., Hossain M. (2011). Migration and health: a framework for 21st century policy-making. PLoS Med.

[5] Editorial. Migration and health. The lancet infectious diseases. 2016;16(8):867. Available at: doi: 10.1016/S1473-3099(16)30218-3. 27477965

[6] Mukumbang F.C. (2020). Are asylum seekers, refugees and foreign migrants considered in the COVID-19 vaccine discourse?. BMJ Glob Health.

[7] Clayton J. More than one million refugees travel to Greece since 2015. United Nations High Commissioner for Refugees. 2016. Available from https://www.unhcr.org/uk/news/latest/2016/3/56e9821b6/million-refugees-travel-greece-since-2015.html [Accessed 9th February 2021]

[8] European Council. EU-Turkey Statement, 18th March 2016. Available at: https://www.consilium.europa.eu/en/press/press-releases/2016/03/18/eu-turkey-statement/ [Accessed 27th March 2021]

[9] International Organisation for Migration. Supporting the Greek authorities in managing the national reception system for asylum seekers and vulnerable migrations (SMS), Factsheets. November 2020. Available at: https://greece.iom.int/sites/default/files/__Merged%20Mainland%20Nov_20_compressed_0_0.pdf [Accessed 9th February 2021]

[10] United Nations High Commissioner for Refugees. Greece Accommodation update. August 2020. Available at: https://data2.unhcr.org/en/documents/details/78795. [Accessed 2nd April 2021]

[11] United Nations High Commissioner for Refugees. Greece factsheet december 2020. Available from https://data2.unhcr.org/en/documents/details/84481 [Accessed 9th February 2021]

[12] International Organisation for Migration. Supporting the Greek authorities in managing the national reception system for asylum seekers and vulnerable migrations (sms), factsheets. November 2020. Available at: https://greece.iom.int/sites/greece/files/__Merged%20Mainland%20Nov_20_compressed_0_0.pdf [Accessed 2nd April 2021]

[13] United Nations High Commissioner for Refugees. Aegean Islands weekly snapshot 16-22 November 2020. Available at: https://reliefweb.int/sites/reliefweb.int/files/resources/GRC_AegeanIslands_WeeklySnapshot_20201122.pdf [Accessed 2nd April 2021]

[14] Human Rights Watch. Greece: Island camps not prepared for Covid-19. April 2020. Available at: https://www.hrw.org/news/2020/04/22/greece-island-camps-not-prepared-covid-19 [Accessed 9th February 2021]

[15] Commentary (2020). Leave no one behind” and access to protection in the Greek Islands in the COVID-19 Era. Int Migr.

[16] Carruthers E, Veizis A, Kondilis E, McCann S. Situational brief: asylum seekers and refugees in Greece during COVID-19 –22 September 2020 – Update 2. London, UK: Lancet Migration and Health; 2020. Available at: https://1bec58c3-8dcb-46b0-bb2a-fd4addf0b29a.filesusr.com/ugd/188e74_3653f716fa4748cca1c52d154c3e86b3.pdf?index=true [Accessed 10th February 2021]

[17] Hargreaves S., Kumar B.N., McKee M., Jones L., Veizis A. (2020). Europe's migrant containment policies threaten the response to covid-19. Br Med J.

[18] Kondilis E., Puchner K., Veizis A., Papatheodorou C., Benos A. (2020). Covid-19 and refugees, asylum seekers, and migrants in Greece. Br Med J.

[19] European Centre for Disease Prevention and Control. Guidance on infection prevention and control of coronavirus disease (COVID-19) in migrant and refugee reception and detention centres in the EU/EEA and the United Kingdom. 15 June 2020. Stockholm: European Centre for Disease Prevention and Control. Available at: https://www.ecdc.europa.eu/sites/default/files/documents/COVID-19-guidance-refugee-asylum-seekers-migrants-EU.pdf [Accessed 10th February 2021]

[20] Fouda A., Mahmoudi N., Moy N., Paolucci F. (2020). The COVID-19 pandemic in Greece, Iceland, New Zealand, and Singapore: health policies and lessons learned. Health Policy Technol.

[21] R. Maltezou. Greece orders nationwide lockdown to curb COVID-19 surge. 5 November 2020. Available from https://www.reuters.com/article/health-coronavirus-greece/update-4-greece-orders-nationwide-lockdown-to-curb-covid-19-surge-idUSL8N2HR3UZ [Accessed on 9th February 2021]

[22] National Public Health Organisation (2020).

[23] National Public Health Organization (2020).

[24] Kondilis E., Papamichail D., Gallo V., Benos A. (2021). COVID-19 data gaps and lack of transparency undermine pandemic response. J Public Health.

[25] Carruthers E., Veizis A., Kondilis E., McCann S. (2020).

[26] National Public Health Organisation. Press conference. 3rd April 2020. Available at: https://eody.gov.gr/wp-content/uploads/2020/04/0403_briefing_covid19.pdf [Accessed 10th February 2021]

[27] Hellenic Government. Legislation for COVID-19 2020. Available at: https://covid19.gov.gr/nomothesia-gia-ton-covid-19/ [Accessed December 20, 2020].

[28] Hellenic Ministry of National Defence. Weekly media reports 2020. Available at: https://geetha.mil.gr/evdomadiaies-anakoinoseis-typoy-etoys-2020/ [Accessed December 20, 2020]

[29] International Organisation for Migration. Supporting the Greek authorities in managing the national reception system for asylum seekers and vulnerable migrants, SMS Factsheets 2020. Available at: https://greece.iom.int/en/sms-factsheets [Accessed December 20, 2020]

[30] Hellenic Statistical Authority. General population census 2011. Available at: https://www.statistics.gr/2011-census-pop-hous [Accessed 11th February 2021]

[31] National Public Health Organisation (2020).

[32] National Public Health Organization (2020).

[33] Dean AG, Sullivan KM, Soe MM. OpenEpi: open source epidemiologic statistics for public health, Version. www.OpenEpi.com, updated 6th April 2013, [Accessed 8 January 2021]

[34] Jalout M. Finding solutions to Greece's refugee education crisis: a Theirworld Report. April 2020. Available at: https://reliefweb.int/sites/reliefweb.int/files/resources/RefugeeEducation-Report-240420-2.pdf [Accessed 2nd April 2021]

[35] Reuters. Greece to ship hundreds of migrants to mainland from camp on Lesbos. 3rd May 2020. Available at: https://www.reuters.com/article/us-health-coronavirus-greece-migrants-idUSKBN22F0Q7 [Accessed on 2nd April 2021]

[36] General Secretariat for Information and Communication. National situational picture regarding the Islands at Eastern Aegean Sea (15/11/2020). 16th November 2020. Available at: https://infocrisis.gov.gr/11141/national-situational-picture-regarding-the-islands-at-eastern-aegean-sea-15-11-2020/?lang=en [Accessed 10th February 2021]

[37] Human Rights Watch. Greece/EU: bring Moria Homeless to Safety. 16th September 2020. Available at: https://www.hrw.org/news/2020/09/16/greece/eu-bring-moria-homeless-safety [Accessed 10th February 2021]

[38] MacGregor M. Moria 2.0: the new Lesbos refugee camp. Infomigrants. 12th October 2020. Available at https://www.infomigrants.net/en/post/27851/moria-2-0-the-new-lesbos-refugee-camp [Accessed 10th February 2021]

[39] MacGregor M. Lesbos camp: asylum seekers fend for themselves as winter looms. Infomigrants. 25th November 2020. Available at: https://www.infomigrants.net/en/post/28724/lesbos-camp-asylum-seekers-fend-for-themselves-as-winter-looms . [Accessed 10th February 2021]

[40] Médecins Sans Frontières. Negligent and dangerous COVID-19 response in Vathy camp, Samos. 26th October 2020. Available at: https://www.msf.org/greece-negligent-covid-19-response-vathy-camp-samos [Accessed 10th February 2021]

[41] Human Rights Watch. Greece: island Camps Not Prepared for COVID-19. April 2020. Available at: https://www.hrw.org/news/2020/04/22/greece-island-camps-not-prepared-covid-19 [Accessed 9th February 2021]

[42] Eikemo T., Avrami L., Cavounidis J., Mouriki A., Gkiouleka A., McNamara C.L. (2018). Health in crises. Migration, austerity and inequalities in Greece and Europe: introduction to the supplement. Eur J Public Health.

[43] United Nations High Commissioner for Refugees. Updated Data of COVID-19 Risk Groups in the Island RICs. 30th November 2020.

[44] Enikos GR. President of POEDIN at enikos.gr: what it reveals about the patients with coronavirus in the hospitals of Attica - How many are from structures. 17th September 2020. Available at: https://www.enikos.gr/society/740044/proedros-poedin-sto-enikosgr-apo-domes-schedon-oi-misoi-astheneis [Accessed 10th February 2021]

[45] Hellenic Ministry of Health. 99 empty ICU beds in Attica, during SYRIZA in 2018 there were 35 people on the waiting list. Press Release 21st September 2020. Available from https://www.moh.gov.gr/articles/ministry/grafeio-typoy/press-releases/7678-b-kikilias-99-kenes-klines-meth-sthn-attikh-epi-syriza-to-2018-htan-35-atoma-sth-lista-anamonhs. [Accessed 9th February 2021]

[46] Cossé E. Human Rights Watch. Greece again extends covid-19 lockdown at refugee camps. 12 June 2020 Available at: https://www.hrw.org/news/2020/06/12/greece-again-extends-covid-19-lockdown-refugee-camps [Accessed 10th February 2021]

[47] World Health Organization, Europe. Interim guidance for refugee and migrant health in relation to COVID-19 in the WHO European Region. 25th March 2020.

[48] The Health System Response Monitor (HSRM). COVID-19: health system response monitor - Malta. Available at: https://www.covid19healthsystem.org/countries/malta/countrypage.aspx [accessed 13th February 2021 ].

[49] The Guardian. Refugees in German centre fear lack of protection as Covid-19 cases soar. 15 April 2020. Available at: https://www.theguardian.com/world/2020/apr/15/refugees-in-german-centre-fear-lack-of-protection-as-covid-19-cases-soar. [Accessed 13th February 2021].

[50] Central Agency for the Reception of Asylum Seekers (COA). Uitkomst testen bewoners en medewerkers azc Sneek. The Hague: COA; 2020. Available at: https://www.coa.nl/nl/actueel/nieuws/uitkomst-testen-bewoners-en-medewerkers-azc-sneek [Accessed 13th February 2021].

[51] D'Ignotic S. Italy's Lampedusa: back on the migration front line. New Humanitarian. Available at: https://www.thenewhumanitarian.org/news-feature/2020/08/31/Italy-Lampedusa-migration [Accessed 13th February 2021].

[52] I.O.M. OHCHR, UNHCR and WHO. The rights and health of refugees, migrants and stateless must be protected. 31st March 2020. Available at: https://www.iom.int/news/rights-and-health-refugees-migrants-and-stateless-must-be-protected-covid-19-response [Accessed 10th February 2021].

[53] Orcutt M., Patel P., Burns R. (2020). Global call to action for inclusion of migrants and refugees in the COVID-19 response [published correction appears in Lancet. 2020 Apr 27;]. Lancet.

[54] Orcutt M., Mussa R., Hiam L., Veizis A., McCann S., Papadimitriou E., Ponthieu A., Knipper M. (2020). EU migration policies drive health crisis on Greek Islands. Lancet.

[55] Gunst M., Jarman K., Yarwood V., Rokadiya S., Capsaskis L., Orcutt M., Abbara A. (2019). Healthcare access for refugees in Greece: challenges and opportunities. Health Policy.

[56] Nezafat Maldonado B.M., Collins J., Blundell H J., Singh L. (2020). Engaging the vulnerable: a rapid review of public health communication aimed at migrants during the COVID-19 pandemic in Europe. J Migr Health.

[57] M. Milenkovski Serbia vaccinates refugees against COVID-19. 30th March 2021. Available at: https://www.unhcr.org/uk/news/stories/2021/3/60632be44/serbia-vaccinates-refugees-against-covid-19.html [Accessed 1st April 2021]

[58] Inter-Agency Standing Committee. Interim guidance public health and social measures for COVID-19 prepardness and response operations in low capacity and humanitarian settings. 2020. Available at: https://interagencystandingcommittee.org/system/files/2020-11/IASC%20Interim%20Guidance%20on%20Public%20Health%20Measures%20for%20COVID-19%20in%20Low%20Capacity%20and%20Humanitarian%20Settings_0.pdf [Accessed 1st April 2021]

[59] UN Women, Translators without borders. COVID-19: how to include marginalized and vulnerable people in risk communication and community engagement. 2020. Available at: https://interagencystandingcommittee.org/covid-19-how-include-marginalized-and-vulnerable-people-risk-communication-and-community-engagement [Accessed 1st April 2021]

[60] Inter Agency Standing Committee. Interim guidance to scaling-up covid-19 outbreak in readiness and response operations in camps and camp-like settings. 2020. Available at: https://interagencystandingcommittee.org/system/files/2020-11/IASC%20Interim%20Guidance%20on%20COVID-19%20for%20Outbreak%20Readiness%20and%20Response%20Operations%20-%20Camps%20and%20Camp%20-%20like%20Settings.pdf [Accessed 1st April 2021]

